# Food insecurity and mental health of college students in Lebanon: a cross-sectional study

**DOI:** 10.1017/jns.2022.68

**Published:** 2022-08-23

**Authors:** Rita Itani, Lama Mattar, Samer Kharroubi, Tania Bosqui, Marwa Diab-El-Harake, Lamis Jomaa

**Affiliations:** 1 Food Security Program, Faculty of Agricultural and Food Sciences, American University of Beirut, Beirut, Lebanon; 2 Nutrition Program, Department of Natural Sciences, Lebanese American University, Beirut, Lebanon; 3 Department of Nutrition and Food Sciences, Faculty of Agricultural and Food Sciences, American University of Beirut, Beirut, Lebanon; 4 Department of Psychology, Faculty of Arts and Sciences, American University of Beirut, Beirut, Lebanon; 5 Department of Human Sciences, College of Health and Sciences, North Carolina Central University, Durham, NC 27707, USA

**Keywords:** College students, Food (in)security, Lebanon, Mental health, Well-being

## Abstract

The present study aimed to assess the prevalence of food insecurity (FI) among college students and explore its association with indicators of mental and psychosocial health. Data were collected using a cross-sectional online survey from college students in different universities in Lebanon during the Spring 2021 semester. FI was assessed using the validated eight-item food insecurity experience scale. The mental health of college students was assessed using validated screening tools for depression, anxiety and well-being, namely the Patient Health Questionnaire (PHQ-9), the General Anxiety Disorder-7 (GAD-7) and the World Health Organization (WHO-5) index, respectively. Multiple linear regression models were conducted to assess the relationship of FI with PHQ-9, GAD-7 and WHO-5 scores. A total of 745 students completed the online survey. Approximately 39 % of students in the sample were experiencing FI of which 27·4, 8·1, and 3·5 % were experiencing mild, moderate and severe FI , respectively. Low maternal education, low household monthly income and high levels of stress were significant correlates of FI among college students (*P*-trend < 0·001). In addition, 22·6 and 34·4 % of students showed severe symptoms of depression and anxiety, respectively. Regression models showed that FI was associated with higher scores on PHQ-9 and GAD-7 (*β* = 2·45; 95 % CI [1·41, 3·49]) and (*β* = 1·4; 95 % CI [1·1, 2·2], respectively) and lower scores on WHO-5 (*β* = −4·84; 95 % CI [−8·2, −1·5]). In conclusion, a remarkable proportion of college students reported experiencing different forms of FI, which was associated with poorer mental health and well-being outcomes. Public health programmes and interventions are needed to mitigate FI and improve student health-related outcomes.

## Introduction

The transition from high school to university encompasses various prospects and challenges among young adults aged between 18 and 25 years old. College students are simultaneously challenged with various responsibilities at the academic, financial and psychosocial levels as they embark into an unfamiliar environment embedded with new experiences^(1,2)^. Such challenges may impact the overall university experience of college students^(1)^.

Food insecurity (FI) is a growing concern among college students^(3)^. FI is defined as the inadequate physical, economic or social access to safe and nutritious food that meets the preferences and dietary needs of individuals^(4)^. A recent systematic review of studies conducted across college campuses in the United States showed that the prevalence of FI among college students in the US was found to be alarming, ranging between 35 and 42 %^(3)^. In parallel, there has been also a significant rise in the prevalence of mental health disorders (25·8 %) among college students in high and low- to middle-income countries (LMICs)^(5,6)^. With the onset of the COVID-19 pandemic, a global increase of 50 million and 76 million cases of depression and anxiety were recorded^(7)^. Women and young adults were the most affected and triggered with the pandemic^(7)^. Similarly, the rates of FI among college students increased by an estimate of one-third during 2020^(8)^.

FI is a multifaceted phenomenon that has been shown to be associated with poor physical, mental and psychosocial health among college students^(9,10)^. Previous studies have shown that food-insecure college students demonstrated higher odds of stress, deteriorated quality of sleep, disrupted eating patterns and behaviours and lower academic performance in comparison to food secure students^(2,11–17)^. FI in all its degrees (marginal, moderate and severe) were also previously shown to be associated with emotional and psychosocial distress among college students^(3,17)^. Despite the growing interest in exploring FI among college students and its impact on various measures of well-being; there remains a dearth of evidence on FI among college students and young adults in low- to middle-income countries.

Lebanon is a small middle-income country in the Middle East & North Africa (MENA) region that continues to struggle with protracted internal, spill-over and external conflicts with devastating economic, political and social repercussions. The country's checkered history and ongoing crises have had serious repercussions on the food security^(18)^ and mental health and well-being of its populations^(19)^. FI is a major public health concern in Lebanon, even before the start of the recent economic and health crises. According to a study conducted in 2015 on a nationally representative sample of Lebanese households with children, authors found that more than 42 % of respondents were experiencing moderate to severe FI^(18)^. Another study conducted by Maalouf *et al.* showed that the prevalence of mental health disorders among adolescents and young adults (aged between 18 and 25 years old) reached 26·1 % which was slightly higher than the average prevalence of such disorders among the Lebanese population (25 %)^(19)^. In 2020, the food security status of the country and its population were further threatened with the repercussions of multiple compounded crises including the COVID-19 pandemic, the tragic Beirut Port blast and an unprecedented economic crisis. The prevalence of poverty and extreme poverty in Lebanon increased from 28 and 8 % in 2019 to reach 55 and 23 %, respectively by 2020^(20)^. In parallel, an alarming rate of mental health disorders with anxiety have been reported among young adults with anxiety reaching approximately 17 %^(19,21)^.

Previous studies showed that college students in Lebanon may be at risk of mental health disorders due to a myriad of stressors related to family, university, country status, financial challenges^(22)^. Yet, to date, only one study explored the experience of FI among college students in Lebanon, whereby authors reported that 10 % of the study sample of college students, who were recruited from one of the branches of the public Lebanese university, were experiencing some form of FI^(23)^ during the COVID-19 pandemic. Nevertheless, the study by Fares and colleagues^(23)^ did not explore the psychosocial and mental health status of college students or the potential associations between FI and mental health parameters.

Thus, the present study aims to address this research gap in Lebanon. The country has been witnessing multiple crises taking a toll on the FI and mental health of its population, including young adults. The specific objectives of the study are to (1) evaluate the experience of FI among a sample of college students in Lebanon, (2) examine the socio-demographic and economic factors associated with FI among college students within the study sample and (3) explore the association between FI status and indicators of mental and psychosocial health among college students in Lebanon.

## Methods

### Study design and sampling

A cross-sectional study was conducted using a convenience sample of college students (aged between 18 and 24 years old) enrolled in public and private universities across Lebanon during the spring semester of 2021 (February–April).

Sample size calculations were conducted to meet the primary and secondary objectives of the study^(24)^. First, a minimum sample of 139 college students was needed to estimate the prevalence of FI in the present study using estimates from a recent study conducted among college students in Lebanon (10 %)^(23)^, while considering a 95 % confidence interval (i.e., 5 % margin of error)^(24)^. Second, power analysis showed that a minimum sample of 634 students was required to explore associations between FI and mental health outcomes in the present study. This was based on estimates from a multi-institutional study that showed increased odds of stress among food-insecure participants (OR = 4·5 [95 % CI 2·66, 8·11]) using a 95 % confidence interval and precision level of 40 %^(2)^. The final intended sample for the present study was 730 college students accounting for an additional 15 % due to incomplete responses.

### Study population and recruitment

This study was conducted according to the guidelines laid down in the Declaration of Helsinki and all procedures involving research study participants were approved by the Institutional Review Boards at the American University of Beirut (AUB) and Lebanese American University (LAU).

All student participants provided their consent to participate in the study through completing an online consent form. Recruitment was conducted through two approaches. The first approach was through sharing the survey with interested and eligible participants through various social media platforms (e.g. Instagram, Facebook, Twitter, etc.). The second approach included sending email invitations to college students enrolled during the spring 2021 semester within two private universities in the country, namely AUB and LAU, to participate in our research study. Email invitations were sent through the HRPP/IRB (Human Research Protection Program/Institutional Review Board) office and non-administrative level employees through the Dean of Student Affairs Office, at the respective universities, to avoid any undue influence or coercion.

### Survey/instruments

The survey was chosen to be administered online given that the country was in a state of total lockdown due to the resurgence of cases of COVID-19 among the general population. College students who accepted to participate in the study were prompted to complete the consent form before the start of the online survey. The online survey included questions on socio-demographic characteristics and lifestyle habits, food security status, psychosocial well-being and mental health of students. Further details regarding the various subsections of the survey are presented below.

#### Socio-demographic characteristics and lifestyle habits

Data on the demographic and socioeconomic characteristics of the study sample were addressed through a total of 19 questions. Students were asked to report their gender, age, university, class level, major, residence, grade point average (GPA), personal income, household monthly income, parental education, parental employment and financial aid status. In addition, university type was collected as part of the survey including renowned private universities in the country such as AUB, LAU, Beirut Arab University (BAU), University of Balamand (UOB) and the University of Saint Joseph (USJ) as well as the Lebanese University (the only public university with multiple branches and majors across the country). Study major was also collected and then reclassified into health- and non-health-related majors. Moreover, students identified their class level (freshmen, sophomore, junior, senior and graduate), and this variable was further re-grouped into undergraduate and graduate levels. For residence, students were asked to select from three items (alone, roommates and family). For parental education, students were asked to choose from four items (Elementary or less, Intermediate, High school and Graduate). Such items were further re-grouped into three groups (Intermediate, High School and University Degree of Higher). Similarly, for parental employment, three groups (Not working, employed part-time and employed full-time) were re-grouped into two groups (Not employed and employed). For household monthly income, students were given nine items to select their monthly income; items were further grouped into four groups (<2 000 000 LBP, 2 000 000–5 000 000 LBP, >5 000 000 LBP and refuse to answer).

Furthermore, six questions were used to collect information on lifestyle habits. Students were asked to determine their average sleeping hours per day, quality of sleep (regular or irregular), average studying hours per day, physical activity level, smoking status (never smoked, occasional smoker, current smoker, ex-smoker, refuse to answer) and perceived stress (scale of 1–10). Students were asked to report on the perceived intensity of stress in their lives from 1 (not stressful at all) to 10 (extremely stressful).

#### Food insecurity status

FI was measured using the Food Insecurity Experience Scale (FIES), an experience-based measure that was developed and validated by the Food and Agriculture organization (FAO) ‘Voices of the Hungry (VOH)’ project^(25)^. The FIES is a reliable, valid and internally consistent indicator with an adequate Cronbach's alpha coefficient of 0·759^(26)^. According to a study conducted by SheikhOmar *et al.*^(27)^, FI was considered a valid indicator to assess FI among Arab countries, such as Lebanon. The tool consists of an eight-point scale assessing people's actual experiences in accessing food. The FIES tool was administered in its validated English and Arabic versions in the present study. Respondents were asked whether, at any time during the previous 12 months, they have worried about their ability to obtain enough food, their household has run out of food or they have been forced to compromise the quality or quantity of the food that they ate owing to limited availability of money or other resources to obtain food. Respondents were assigned a score value of ‘1’ for any specific question that they have answered ‘yes’ and ‘0’ if their answer was ‘no’. For each respondent, the assigned values for the eight questions were summed to obtain a raw score that ranges from 0 to 8. The total score was used to classify individual-level FI status: food secure (raw scores = 0); mild FI (1–3); moderate FI (4–6) and severe FI (7–8)^(25)^. For analyses, FI was further recoded into two categories: 1 – food secure (raw score = 0) and 2 – food insecure (raw score ≥ 1); the latter included individuals experiencing mild, moderate or severe FI.

#### Psychosocial well-being and mental health

The psychosocial well-being and mental health of college students in the present study were assessed using the following screening indicators: Patient Health Questionnaire (PHQ-9), General Anxiety Disorder-7 (GAD-7) and the World Health Organization (five) Index (WHO-5).

Patient Health Questionnaire (PHQ-9) is a 9-item self-reported depression scale and it is one of the most validated tools in screening for mental health^(28)^. The PHQ-9 is an internally consistent and reliable indicator among college students with a Cronbach's alpha coefficient >0·8 in English and its Arabic versions^(29,30)^. PHQ-9 tool was previously used in Lebanon, and it was found to be a valid indicator to screen for depression^(31)^. Students in the study sample were asked how frequently they have been feeling sad or depressed, tired, or having little to no energy, etc., during the last 2 weeks (e.g. not all, several days, more than half of the days, nearly every day). The response to each item was graded on the Likert scale indicating 0 ‘absence of a symptom’ to 3 ‘presence of symptom nearly every day’. For each respondent, the assigned values for items of the PHQ-9 tool were all summed to obtain a raw score that ranges from 0 to 27. The total PHQ-9 score was used to classify individuals attributing to minimal (0–4), mild (5–9), moderate (10–14), moderately severe (15–19) and severe depression (20 or greater), respectively^(28)^.

General Anxiety Disorder-7 (GAD-7) is a 7-item self-reported anxiety scale. GAD-7 was also previously validated tool in the local context, and it was found to be an efficient and adequate tool to screen for general anxiety disorder and thus, assess its level of severity across the Lebanese population^(31)^. The scale was reported as an acceptable and reliable indicator among college students with a Cronbach's alpha coefficient >0·7 in English and its Arabic versions^(30,32)^. Students in the present study were asked if they have been nervous or anxious or on edge, not being able to stop or control worrying, having trouble relaxing, worrying too much about different things, restless, easily irritated, and annoyed, afraid that something might happen. Students then selected a time-frequency of experiencing such episodes whether not all, several days, more than half of the days, nearly every day which were given scores of 0, 1, 2 and 3, respectively. The total scores of GAD-7 ranged from 0 to 21 with 0–4, 5–9, 10–14 and 15–21 representing minimal anxiety, mild anxiety, moderate anxiety and severe anxiety^(31)^.

The 5-item World Health Organization Well-Being Index (WHO-5) is one of the most frequently used questionnaires to assess overall subjective well-being. It is a validated and internally consistent tool with a Cronbach alpha of 0·83 and 0·85 in its Arabic and English versions, respectively^(33,34)^. WHO-5 presents a 5-items related scheme to feelings of cheerfulness and good spirits, calmness and relaxation, activeness and vigorousness, freshness, and restfulness^(33)^. Students in the present study were asked to denote a time-frequency of experiencing such a series of episodes, i.e. at no time, some of the time, less than half of the time, more than half of the time, most of the time and all of the time which denote a score of 0, 1, 2, 3, 4 and 5, respectively. The total score of WHO-5 ranged from 0 to 25 and was then multiplied by 4 to provide a final score ranging from 0 which represents diminished being to 100 representing optimal well-being^(33)^.

### Statistical analyses

Data were analysed using the Statistical Package for the Social Sciences (SPSS) version 25.0. Descriptive statistics were presented as means and standard errors (SEs) for continuous variables and as proportions and frequencies for categorical variables. Normality tests were carried out for all variables. Chi-square tests and independent *t*-tests were used to examine the associations between FI and the socio-demographic characteristics and lifestyle factors of college students. First, socio-demographic and lifestyle correlates of FI were explored using simple and multiple logistic regression analyses. The dependent variable in these models was FI status, a dichotomised variable, which was recoded into two categories: 1 – food secure and 2 – food insecure (mildly, moderately and severely food insecure individuals). Second, to further examine the associations between FI with mental health indicators (continuous outcome variables), simple and multiple linear regression analyses were conducted. More specifically, simple linear regression models were used to examine the associations between FI (independent variable) with PHQ-9, GAD-7 and WHO-5 scores, respectively, as dependent continuous outcomes. Socio-demographic and lifestyle variables found significantly associated with the dependent variables were adjusted for in all multiple regression models. Results from the logistic regression models were expressed as odds ratio (OR) with 95 % confidence intervals (CIs). Results from the linear regression models were expressed as Beta coefficients (*β*) with 95 % CI. All reported *P*-values were based on two-sided tests and were compared with a significance level of 5 %.

## Results

### Food insecurity status and socio-demographic characteristics of participants

A total of 760 students filled the online survey of which 745 had complete data and were considered in our final analysis (98 % completion rate). Table 1 presents the socio-demographic characteristics and lifestyle behaviours of college students in the study sample. In brief, the average age of college students was 20·84 (se = 0·2) years. Most students were enrolled in private universities (85·4 %) and identified as females (64·0 %) (Table 1). Other sociodemographic characteristics and lifestyle behaviors of college students in the study sample were presented in Table 1. As shown in Table 2, significant differences were observed in socio-demographic characteristics, and lifestyle behaviours of college students by food security status. Results from the simple logistic regression analyses showed that FI was significantly associated with maternal education and employment, household monthly income, foreign currency, financial aid/loan/scholarship (no payment required), average sleeping hours/day and level of stress (Table 2). However, the multiple logistic regression model showed that college students who had mothers with higher educational levels or those with a high monthly household income (greater than 5 000 000 LBP) were 52% and 74 % less likely to be food insecure, respectively, compared to their counterparts (OR = 0·48; 95 % CI [0·27, 0·86] and OR = 0·26; 95 % CI [0·15, 0·4], respectively). Furthermore, as the stress level among college students increases, the odds of FI increased by 25 % compared to food secure students (OR = 1·25; 95 % CI [1·14, 1·37] (Table 2).

**Table 1.** Socio-demographic characteristics and lifestyle factors of college students in Lebanon in the study sample (*n* 745), 2021

Socio-demographic characteristics	*N* or Mean	%^d^ or se
Gender		
Male	223	30·0
Female	479	64·0
Non-binary^a^	43	6·0
Age (years)	20·84	0·2
Nationality		
Lebanese	653	90·1
Non-Lebanese	72	9·79
University^b^		
Public	106	14·6
Private	618	85·4
Class level		
Undergraduate	509	71·2
Graduate	206	29·0
Major		
Non-Health-related	471	64·0
Health-related	263	36·0
Residence		
Alone	52	7·3
Family	588	82·6
Roommates	72	10·1
Father's educational level		
Intermediate or less	128	18·0
High School	166	22·5
University Degree or Higher	416	56·3
Mother's educational level		
Intermediate or less	89	12·3
High School	227	31·3
University Degree or Higher	408	56·4
Father's employment status		
Not employed	162	23·0
Employed/Self-employed	538	72·2
Mother's employment status		
Not employed	268	37·0
Employed/Self-employed	461	63·0
Household monthly income (LBP)^c^		
<2 000 000 (<250 USD)	192	28·2
2 000 000–5 000 000 (250–625 USD)	161	23·6
>5 000 000 (>625 USD)	149	22·0
Refuse to answer	179	26·3
Earnings from income as Foreign Currency		
Yes	117	15·7
No	506	68·1
Average student personal income/month (LBP)	530 000	47 630
Student current job		
Unemployed	551	77·0
Employed	165	23·0
Financial aid/loan/scholarship (Not payment required)		
Yes	379	58·3
No	271	41·7
Financial aid/loan/scholarship (Payment required)		
Yes	114	18·7
No	495	81·3
Current GPA (out of 4)	3·5	0·12
Expected GPA (out of 4)	3·72	0·17
Food security status		
Food secure	455	61·0
Food insecure	290	39·0
Lifestyle factors		
Average sleeping hours per day		
<7 h	163	22·4
≥7 h	565	77·6
Description of sleeping habits		
Irregular	326	45·2
Regular	396	54·8
Average studying hours per week	23·35	0·73
Average level of stress (1–10)	6·7	0·08

a Non-binary individuals cannot identify within the margins of gender, i.e. females or males.

b Lebanese University is the only public university in the country. Private Universities included American University of Beirut, Lebanese American University, Beirut Arab University, University of Balamand, Lebanese International University, Modern University of Business and Science.

c 1USD = 8000 LBP. This exchange rate at the time of the study.

d Categorical variables were presented as *n* (%) and continuous variables were presented as means and standard errors (se).

**Table 2.** Simple and multiple logistic regression models examining the association between food insecurity (FI) status and the socio-demographic characteristics and lifestyle factors of college students in Lebanon in the study sample (*n* 745), 2021

Socio-demographic characteristics	Food secure	Food insecure	*P*-value^†^	Unadjusted OR^a^, (95 % CI)	Adjusted OR^b^, (95 % CI)	*P*-value
Gender			0·041*			
Male	124 (27·3)	99 (34·1)		1	1	
Female	299 (65·7)	180 (62·1)		0·75 (0·55, 1·04)	0·821 (0·54 to 1·25)	
Non-binary^c^	32 (7)	11 (3·8)		0·43 (0·21, 0·90)	0·340 (0·09 to 1·16)	0.07
Age (years)	20·9 ± 0·15	20·74 ± 0·2	0·501	0·98 (0·939, 1·031)	−	
Nationality			0·099			
Lebanese	401 (91·6)	252 (87·8)		1	−	
Non-Lebanese	37 (8·4)	35 (12·2)		0·66 (0·408, 1·083)		
University Type^d^			0·013*			
Public	53 (12)	53 (18·7)		1	1	
Private	388 (88)	230 (81·3)		1·70 (1·1, 2·5)	0·80 (0·42, 1·51)	0.71
Class level			0·550			
Undergraduate	304 (70·4)	205 (72·5)		1		
Graduate	128 (29·6)	78 (27·6)		0·90 (0·6, 1·2)	−	
Major			0·230			
Non-Health-related	262 (58·4)	179 (62·8)		1		
Health-related	187 (41·6)	106 (37·2)		0·83 (0·612, 1·125)	−	
Residence			0·171			
Alone	30 (6·9)	22 (8)		Ref		
Family	367 (84·6)	221 (79·5)		0·82 (0·46,1·46)	−	
Roommates	37 (8·5)	35 (12·6)		1·29 (0·63, 2·65)	−	
Father's educational level			0·092			
Intermediate or less	74 (17)	54 (19)		1		
High School	92 (21)	74 (26)		1·10 (0·69,1·76)		
University Degree or Higher	268 (61)	148 (53)		0·76 (0·50, 1·13)		
*P*-trend			0·075			
Mother's educational level						
Intermediate or less	45 (10·2)	44 (15·5)		1	1	
High School	121 (27·4)	106 (37·5)		0·90 (0·559, 1·5)	0·84 (0·45, 1·6)	
University Degree or Higher	275 (62·4)	133 (47)		0·49 (0·31, 0·79)	0·48 (0·27,0·86)	<0.05*
*P*-trend			<0·001***			
Father's employment status			0·053			
Not employed	89 (21·0)	73 (27)		1		
Employed/Self-employed	341 (79·0)	197 (72·9)		0·70 (0·49, 1·01)	−	
Mother's employment status			0·001**			
Not employed	143 (32·0)	125 (44·3)		1	1	
Employed/Self-employed	304 (68·0)	157 (55·7)		0·59 (0·43, 0·80)	0·96 (0·634, 1·444)	0.52
Household monthly income (LBP)^e^						
<2 000 000 (<250 USD)	75 (18·1)	117 (44·3)		1	1	
2 000 000–5 000 000 (250–625 USD)	84 (20·1)	77 (29·1)		0·59 (0·38, 0·90)	0·76 (0·465,1·254)	0.51
>5 000 000 (>625 USD)	114 (27·3)	35 (13·3)		0·20 (0·12, 0·32)	0·26 (0·15, 0·4)	<0.001***
Refuse to answer	144 (34·5)	35 (13·3)		0·16 (0·097,0·249)	0·19 (0·1, 0·35)	<0.001***
*P*-trend			<0·001***			
Earnings from income as Foreign Currency			<0·001***			
Yes	82 (18·1)	35 (12·1)		Ref		
No	278 (61·4)	228 (78·6)		0·52 (*0·34, 0·80)*	0·71 (0·48,1·12)	0.72
Average student personal income/ month (LBP)	570 000 ± 72 144	464 000 ± 46 721	0·278	1·000 (1·000, 1·000)	−	
Student current job			0·014*			
Unemployed	212 (53·8)	167 (65·2)		1	1	
Employed	182 (46·2)	89 (34·8)		0·62 (0·45, 0·86)	0·84 (0·54, 1·30)	0.76
Financial aid/loan/scholarship (No payment required)			0·004**			
Yes	212 (53·8)	167 (65·2)		1	1	
No	182 (46·2)	89 (34·8)		0·62 (0·45, 0·86)	0·84 (0·54, 1·30)	0.90
Financial aid/loan/scholarship (Payment required)			0·456			
Yes	65 (17·8)	49 (20·2)		1		
No	301 (82·2)	194 (79·8)		1·17 (0·77, 1·77)	−	
Current GPA (out of 4)	3·6 ± 0·2	3·3 ± 0·3	0·298	0·93 (0·782, 1·11)	−	
Expected GPA (out of 4)	3·6 ± 0·13	3·2 ± 0·4	0·248	1·02 (0·99, 1·05)	−	
Lifestyle factors						
Average sleeping hours per day			0·042*			
<7 h	88 (19·9)	75 (26·3)		1	1	
≥7 h	355 (80·1)	210 (73·7)		0·69 (0·50,0·99)	0·97 (0·61, 1·55)	0.08
Description of sleeping habits			0·002**			
Irregular	219 (49·8)	107 (37·9)		1		
Regular	221 (50·2)	175 (62·1)		1·62 (1·20, 2·2)	−	
Average studying hours per week	24·3 ± 0·9	22 ± 1·24	0·085	0·99 (0·986,1·00*)*	−	
Average level of stress (1–10)	6·2 ± 0·1	7·44 ± 0·13	<0·001***	1·28 (1·19, 1·38)	1·2 (1·1, 1·4)	<0.05*

a OR (odds ratio) of the dependent variable (food-insecure *v*. food-secure) is presented with 95 % CIs (confidence intervals) using simple logistic regression. The food-insecure category included mildly, moderately and severely food-insecure participants.

b Adjusted ORs are presented with 95 % CIs using multiple logistic regression analysis. The models were adjusted for age and socio-demographic characteristics found to be significant correlates of FI (gender, university type, mother's educational level, mother's employment, household monthly income, foreign currency, financial aid (no payment required, average sleeping hours and level of stress)).

c Non-binary individuals cannot identify within the margins of gender, i.e. females or males.

d Lebanese University is the only public university in the country. Private Universities included American University of Beirut, Lebanese American University, Beirut Arab University, University of Balamand, Lebanese International University, Modern University of Business and Science.

e 1USD = 8000 LBP. This exchange rate at the time of the study.

† *χ*^2^ tests were conducted to determine differences between categorical variables and binary food security status, and independent t-tests were used to determine differences between continuous variables and binary food security status.

*P < 0·05, **P < 0·01, ***P < 0·001.

### Mental health and well-being of college students

Among the study sample, the average score on the PHQ-9 test was 11·94 (se = 0·27) indicating moderate depression. In specific, 69·2 % of students presented with mild to moderately severe symptoms, and 22·6 % showed severe symptoms of depression (see Fig. 1 in Supplementary Material). In terms of GAD-7, the average score was 11·18 (se = 0·24) indicating moderate anxiety. An estimate of 49·4 % presented with mild to moderate symptoms of anxiety, and 34·4 % of students showed severe symptoms of anxiety (see Fig. 2 in Supplementary Material). In terms of overall well-being and on a scale of 0–100, the average score on the WHO-5 was 36·11 (se = 0·87).

Tables 3 and 4 show simple and multiple linear regression models examining the associations between FI and mental health indicators of college students, respectively. Using simple linear regression analysis, FI among college students was associated with higher scores on PHQ-9 and GAD-7 (*β* = 4·2; 95 % CI [3·2, 5·3] and *β* = 3·6; 95 % CI [2·7, 4·5], respectively) and lower scores on WHO-5 in comparison to food secure students (*β* = −11·8; 95 % CI [−15·2, −8·4]) (Table 3). Such associations between FI, PHQ-9, GAD-7 and WHO-5 remained statistically significant even after adjusting for age and other socio-demographic variables (*β* = 2·45; 95 % CI [1·41, 3·49)], *β* = 1·4; 95 % CI [0·53, 2·2] and *β* = −4·8; 95 % CI [−8·2, −1·5], respectively) (Table 4). The adjusted models showed that college students with household monthly incomes (>5 000 000 LBP) had significantly higher well-being (WHO-5) scores compared to college students with the lowest income (*β* = 3·8; 95 % CI [0·07, 7·6]). Furthermore, a one-unit increase in stress level scores among college students was significantly associated with higher depression (PHQ-9) and anxiety (GAD-7) yet lower well-being (WHO-5) scores (*β* = 1·4, 95 % CI [1·23, 1·6], *β* = 1·5, 95 % CI [1·3, 1·7] and β = −4·3; 95 % CI [−5·0, −3·6], respectively). Moreover, students with adequate sleeping hours (>7 h/d) had higher well-being scores on WHO-5 compared to those who had inadequate sleep (*β* = 5·5; 95 % CI [1·7, 9·3] (Table 4).

**Table 3.** Simple regression models examining the associations between food insecurity and mental health indicators of college students in Lebanon aged 18–25 years (*n* 745), 2021^a^

	*β* (95 % CI)					
	Patient Health Questionnaire-9 (PHQ-9)	p-value^b^	General Anxiety Disorder-7 (GAD-7)	p-value^b^	World Health Organization Index-5 (WHO-5)	p-value^b^
Food insecurity	4·2 (3·2, 5·3)	<0.001***	3·6 (2·7, 4·5)	<0.001***	−11·8 (−15·2, −8·4)	<0.001***
Socioeconomic & lifestyle correlates						
Age	−0·2 (−0·40, −0·62)	<0.01**	−0·24 (−0·38, −0·95)	0.001**	0·53 (−0·003, 1·1)	
Gender: Male (Ref)						
Female	−0·84 (−2·01, 0·33)		−0·28 (−1·26, 0·71)		−1·19 (−4·78, 2·40)	
Non-binary	−0·15 (−2·66, 2·36)		1·34 (−0·67, 3·36)		1·15 (−6·30, 8·59)	
University: Public (Ref)						
Private	−1·10 (−2·58, 0·37)		0·04 (−1·307, 1·39)		3·96 (−0·99, 8·91)	
Mother's education level Intermediate or less (Ref)						
High school	1·3 (−0·50, 3·1)		0·36 (−0·65,1·4)		−0·22 (−3·9, 3·5)	
University degree or higher	0·43 (−1·3, 2·1)		0·11 (−0·85, 1·1)		−0·12 (−3·6, 3·4)	
Mother's employment status Unemployed (Ref)						
Employed/Self-employed	−1·1 (−2·2, −0·01)	<0.05*	−0·76 (−1·7, 0·22)		2·4 (−1·2, 5·9)	
Household monthly income (LBP)^c^ <2 000 000 (<250 USD) (Ref)						
2 000 000–5 000 000 (250–625 USD)	−1·6 (−3·1, −0·11)	<0.05*	1·1 (−0·01, 2·3)		−2·6 (−6·8, 1·5)	
>5 000 000 (>625 USD)	−2·5 (−4·0, −0·95)	<0.05*	−0·87 (−2·0, 0·30)		5·7 (1·5, 10·0)	<0.01**
Refuse to answer	−2·0 (−3·5, −0·44)	<0.05*	−1·7 (−2·8, −0·57)	<0.05*	2·4 (−1·7, 6·6)	
Level of stress	1·5 (1·3, 1·7)	<0.001***	1·6 (1·5, 1·8)	<0.001***	−4·9 (−5·6, −4·3)	<0.001***
Average sleeping (≥7 h/d)	−**		−2·8 (−3·9, −1·7)		10·8 (6·8, 14·9)	

The PHQ-9 is a 9-item self-report depression scale that is used to screen and measure the severity of depression. The scoring of the PHQ-9 is obtained by the sum of the scores of the 9 items ranging from 0–37, 0–4, 5–9, 10–15, 15–19 and 20 or greater representing minimal, mild, moderate, moderately severe and severe depression.

The GAD-7 is a 7-item self-report anxiety scale that is used to screen and measure the severity of generalised anxiety disorders. The GAD-7 score is calculated by assigning scores of 0, 1, 2 and 3, to the response categories of ‘not at all’, ‘several days’, ‘more than half the days’ and ‘nearly every day’, respectively, and then adding together the scores for the seven questions. GAD-7 total score for the seven items ranges from 0 to 21. The WHO-5 well-being index measures current well-being. The raw score is calculated by totalling the figures of the five answers. The raw score ranges from 0 to 25, 0 representing worst possible and 25 representing the best possible quality of life. To obtain a percentage score ranging from 0 to 100, the raw score is multiplied by 4.

Average sleeping hours was removed from the PHQ-9 linear regression model as the PHQ-9 test consists of a question related to sleep.

a *β* of the dependent variables (PHQ-9, GAD-7, WHO-5) are presented with 95 % CI using simple linear regression.

b *p<0.05, **p<0.01, ***p<0.001 to indicate significance between variables and mental health indicators.

c 1USD = 8000 LBP. This exchange rate at the time of the study.

**Table 4.** Multiple regression models examining the associations between food insecurity and mental health indicators of college students in Lebanon aged 18–25 years (*n* 745), 2021^a^

	Adjusted β (a*β)* (95 % CI)					
	Patient Health Questionnaire-9 (PHQ-9)	p-value^b^	General Anxiety Disorder-7 (GAD-7)	p-value^b^	World Health Organization Index-5 (WHO-5)	p-value^b^
Food insecurity	2·45 (1·41, 3·49)	<0.001***	1·4 (1·1, 2·2)	0.001**	−4·8 (−8·2, −1·5)	<0.001***
Socioeconomic & lifestyle correlates						
Age	−0·15 (−0·27, −0·06)	<0.05*	−0·20 (−0·32, −0·09)	0.001**	−	
Mother's employment status Unemployed (Ref)						
Employed/Self-employed	−0·20 (−1·2,0·814)		−		−	
Household monthly income (LBP)^c^ <2 000 000 (<250 USD) (Ref)						
2 000 000–5 000 000 (250–625 USD)	−1·27 (−2·5, 0·14)		−		−	
>5 000 000 (>625 USD)	−1·4 (−2·8, 0·03)		−		3·82 (0·07, 7·6)	
Refuse to answer	−0·36 (−1·73, 1·01)		−0·99 (−1·9, −0·06)	<0.05*	−	
Level of stress	1·4 (1·23 1·6)	<0.001***	1·5 (1·3, 1·7)		−4·3 (−5·0, −3·6)	<0.001***
Average sleeping (≥7 h/d)	−		−0·86 (−1·8, 0·09)		5·5 (1·7, 9·3)	<0.01**

The PHQ-9 is a 9-item self-report depression scale that is used to screen and measure the severity of depression. The scoring of the PHQ-9 is obtained by the sum of the scores of the nine items ranging from 0–37, 0–4, 5–9, 10–15, 15–19 and 20 or greater representing minimal, mild, moderate, moderately severe and severe depression.

The GAD-7 is a 7-item self-report anxiety scale that is used to screen and measure the severity of generalised anxiety disorders. The GAD-7 score is calculated by assigning scores of 0, 1, 2 and 3, to the response categories of ‘not at all’, ‘several days’, ‘more than half the days’ and ‘nearly every day’, respectively, and then adding together the scores for the seven questions. GAD-7 total score for the seven items ranges from 0 to 21. The WHO-5 well-being index measures current well-being. The raw score is calculated by totalling the figures of the five answers. The raw score ranges from 0 to 25, 0 representing worst possible and 25 representing the best possible quality of life. To obtain a percentage score ranging from 0 to 100, the raw score is multiplied by 4.

Average sleeping hours was removed from the PHQ-9 linear regression model as the PHQ-9 test consists of a question related to sleep.

a Adjusted *β* (a*β*) are presented with 95 % CI using multiple linear regression analysis. The models were adjusted for socio-demographic characteristics and lifestyle factors found to be significant with these indices (food insecurity, age, gender, mother's education, mother's employment, household monthly income and level of stress).

b *p<0.05, **p<0.01, ***p<0.001 to indicate significance between variables and indicators of mental health and well-being.

c 1USD = 8000 LBP. This exchange rate at the time of the study.

## Discussion

The present study is the first to our knowledge in Lebanon and among the very few conducted in the MENA region^(23,35)^ to examine the prevalence of FI among a large sample of college students and to explore potential associations with mental health and well-being. Our study findings showed that college students in Lebanon are experiencing alarming levels of FI; and the latter was associated with increased depression, anxiety and lower levels of well-being among college students.

The prevalence of FI reported in the present study (39 %) was found to be remarkably higher than that reported earlier in the country among college students from one of the branches of the Lebanese public university (estimated at 10 %)^(23)^. Differences noted with the study conducted by Fares *et al.*^(23)^ may be attributed to methodological discrepancies that include a limited sample size with a focus on a single institution in the latter study. Nonetheless, the prevalence of FI reported in the present study was in line with the projections estimated by authors in another study using the GWP 2015–2017 empirical data for Lebanon^(36)^. According to Kharroubi *et al.*^(36)^, FI was predicted to range between 36 and 39 % by 2022 among the general population with 50–70 % income reduction scenarios due to multiple crises witnessed in the country since 2019. Study findings were also in line with the reported rates of FI among college students presented from other LMICs and high-income countries (HICs), including Iran (44 %)^(35)^, US (35–42 %)^(3)^ and Canada (30 %)^(37)^, respectively.

Our study findings need to be also interpreted within the context of the deteriorating situation in Lebanon. The economic crisis, devaluation of the Lebanese currency and the COVID-19 pandemic have left more than 49 % of the Lebanese population to experience limited access to food with 31 % being unable to eat nutritious food in 2020^(20)^. In addition, the tragic August 4th Beirut port explosion in 2020 along with the demolishion of the major grain silos for Lebanon contributed further to the worsening of the food security situation in the country. Concomitantly, the rates of multidimensional poverty doubled between 2019 and 2021 and reaching 82 % in 2022^(37)^. Such national turmoil could have contributed to the exacerbated FI levels among all population groups, including young adults and college students.

With respect to the correlates of FI, our results showed that FI was associated with maternal education, household income and stress levels, even after adjusting for other socio-demographic and financial factors. Previous studies conducted on college students in Lebanon^(23)^ and Iran^(35)^ have similarly reported low household monthly income as an economic correlate of FI among college students yet these studies were not able to identify any association between maternal education, stress and FI. Nevertheless, our study findings validated the work of previous researchers in the US, Canada and France^(2,38–40)^ that have demonstrated that low parental education, low household income and stress can be strong socioeconomic correlates of FI among college students. Yet, in contrary to other studies published on college students in other settings including Malaysia, the US and Canada^(2,13,39,41,42)^, our study showed no gender-based differences in FI among college students in the study sample. We hypothesize that the lack of association between female sex and the non-binary gender identity with FI within the present study may be explained by the overall looming economic and political challenges that are affecting the status of food security across various subpopulation groups in the country. Nevertheless, this is an area that requires much further exploration given the previously documented gender disparities in FI reported in Lebanon and other countries in the Arab world^(43)^, and the limited research examining the experiences and perceptions of people with different gender identities on their FI and health outcomes.

The alarming depression and anxiety rates reported in the present study were found to be also higher than previous results reported among university students’ pre-collective crises in Lebanon using identical mental health indicators. For example, our study findings showed that almost 70 % of students presented with mild to moderately severe symptoms of depression, and about half of the student sample experienced mild to moderate symptoms of anxiety, whereas results from two studies conducted among university students in Beirut, Lebanon in 2018 showed that 56 % were presenting with mild to moderately severe symptoms of depression, while 36 and 34 % of their study samples showed combined symptoms of depression and anxiety, respectively^(22,44)^. Such differences in trends may be attributed to methodological discrepancies that focused on single institutions in the latter studies^(22,44)^. Another potential explanation may be attributed to the ongoing collective crises such as the COVID-19 pandemic, August 4th Port explosion, political and economic turmoil in the country that may have increased the mental health challenges faced by young adults over the past few years in Lebanon. Resarchers have shown that with the COVID-19 pandemic and the deterioration of the Lebanese economy and political state, young adults are appearing to be negatively impacted by the ongoing crises on multiple physical and mental health levels^(21,45)^. Moreover, the depression and anxiety levels reported in the present study were also significantly higher than those reported among college students in HICs such as the US (12·8 and 15·9 %)^(44)^ and Canada (19 and 32·6 %)^(46)^. Nonetheless, our findings validated the relationship between increased stress and poor mental health among young adults, as previously reported in the scientific literature^(47,48)^.

Upon exploring the associations between FI and mental health parameters through adjusted regression models, our study findings were in accordance with the scientific literature. Results showed that FI is associated with adverse mental and psychosocial health outcomes^(2,3,5,11,17,35,49–53)^. Such association between FI and mental health may appear as bidirectional in nature, and thus, may be explained through various mechanisms. On the one hand, FI may interfere with the psychosocial health and well-being of college students through inducing a series of uncertainty towards the ability to access food that is safe and nutritious as demonstrated in a systematic review conducted by Pourmotabbed and colleagues^(49)^. Hence, food insecure students may show signs and symptoms of psychological distress that include anxiety and depression^(49)^. Furthermore, food insecure students might feel that they are less worthy of adequate living in contrast to their more food secure counterparts^(50)^. Therefore, such a state might impact their mental health and overall well-being. Yet, on the other hand, a deterioration in mental health can lead to an overall decrease in one's productivity, which in turn translates to an increased vulnerability towards FI^(51)^. Regardless of the mechanisms that may explain such associations, the high prevalence of FI and the decline in mental health represent serious threats to the overall health and well-being of college students in the short and long term. The impact of poor mental health and FI among college students induces not only short-term effects such as fatigue, lack of productivity, decreased motivation and poorer academic achievement, but can also have long-term effects. Such adverse effects may lead to an increased risk of mental health disorders along with and increased risk of chronic diseases on the long-run^(9,17,49)^.

### Strengths and limitations

Our present study has several strengths worth highlighting. It is the first study, to our knowledge, that explores the associations between FI and the indicators of mental health and well-being among a diverse group of college students in the region. The FI and mental health indicators used in the present study were previously translated and validated screening tools that were used among similar young adult population in Lebanon^(27,31)^. Another strength of the study is the reasonable duration of the survey to reduce the burden on participants and to avoid any discomfort. Nevertheless, our findings need to be interpreted considering a few limitations. First, the present study follows a cross-sectional design; hence, we were not able to determine the causality of associations between FI and indicators of mental and psychosocial health and well-being among young adults. Second, the survey was completed online given the COVID-19 restrictions at the time of data collection and a convenience sampling approach was adopted in recruitment. Thus, our study findings cannot be generalisable to all university students across the country. Nevertheless, the research team exerted every effort to enhance recruitment of college students from various higher educational universities across the country using multiple social media channels in addition to the email invitations sent to university students. A third limitation that cannot be ruled out is the risk of respondent bias as student may have under- or over-reported their experiences with FI and different measures of mental health and well-being that are used as self-assessment screening tools. Another limitation of the study is the lack of dietary intake data that may have provided a better understanding of the associations between FI, mental health and nutritional status of young adults and that may be worth further examination in follow-up studies.

## Conclusion

The present study sheds the light on the alarming FI, anxiety and depression rates reported among college students in Lebanon. Our findings highlighted the association between FI and poor mental health outcomes among university students, a group that has not been adequately explored in the scientific literature, particularly in the MENA region. The results of our study echo the need for higher educational institutions in Lebanon and similar contexts to consider initiatives and interventions that can help address and mitigate the growing problem of FI on campuses. Universities may consider establishing food banks on campuses or subsidising meals in cafeterias to help alleviate FI while also ensuring that there would be no stigmatisation for those benefiting from such services^(2)^. Such interventions also need to extend beyond university campuses to engage with key decisionmakers and stakeholders across the country including the Ministries of Education and Higher Education, Ministry of Social Affairs, and the Ministry of Health among other governmental and non-governmental entities. Multidisciplinary engagement is crucial to address the ever-increasing needs of various population groups in the country, including youth and young adults. Future studies need to further explore the experiences, perceptions and coping strategies of college students experiencing FI and its repercussions on their health outcomes. In addition, public health and nutrition education interventions need to be considered as potential entry points to help mitigate FI and its repercussions on the food security, health and wellbeing of young adults.

## Supplementary material

For supplementary material accompanying this paper visit https://doi.org/10.1017/jns.2022.68. click here to view supplementary material
