# Supplementary figures and images for: Food insecurity and mental health of college students in Lebanon: a cross-sectional study
Source: J Nutr Sci. 2022 Aug 23;11:e68. doi: 10.1017/jns.2022.68 (PMC9428660; doi:10.1017/jns.2022.68)

**FIGURE 1**

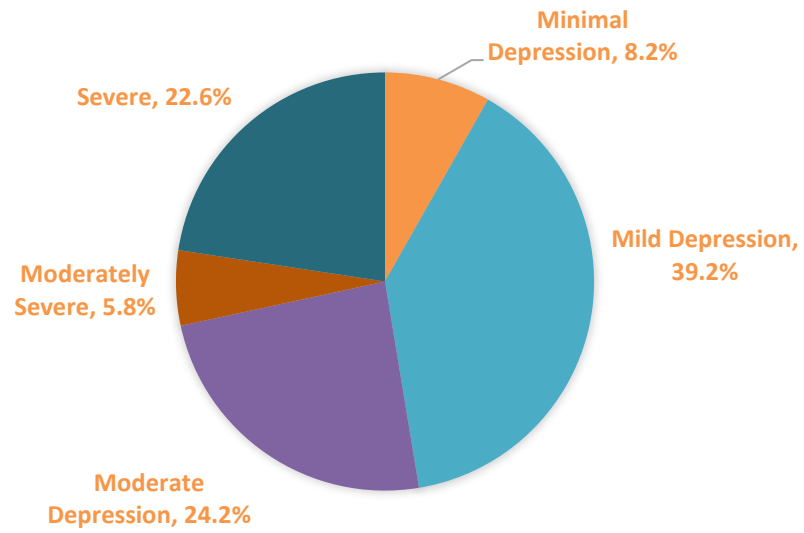

Supplement: Supplementary file 1 [file jnssup.zip › S2048679022000684sup001.pdf]

**FIGURE 2**

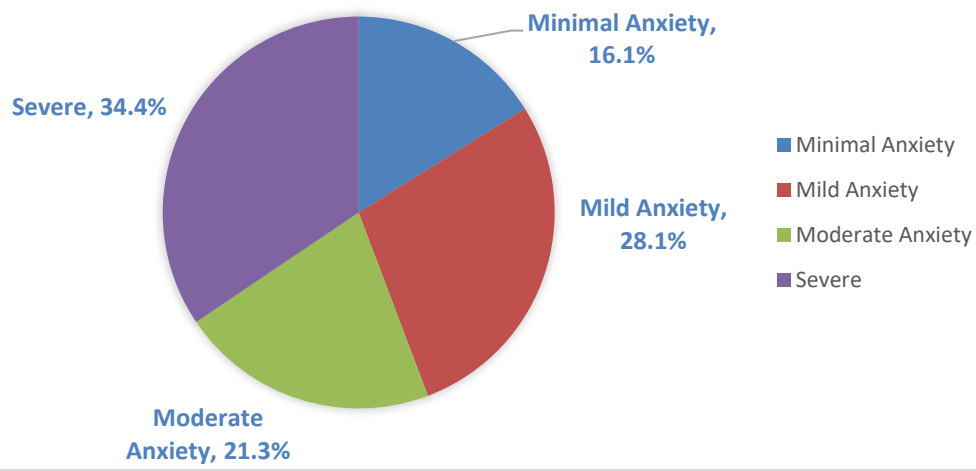

Supplement: Supplementary file 1 [file jnssup.zip › S2048679022000684sup002.pdf]
